# Multifunctional Bioactive Scaffold Facilitating BMSCs‐Driven Osteogenesis and Vascularization in Critical‐Sized Bone Defect Repair

**DOI:** 10.1002/advs.202522692

**Published:** 2026-04-09

**Authors:** Yunze Feng, Bingzhi Chen, Yu Xu, Xin Xu, Bingtao Hu, Tongbo Li, Dingxin Zhang, Baoshuai Bai, Chen Liu, Wanlong Xu, Le Li, Wencan Zhang, Haipeng Si

**Affiliations:** ^1^ Department of Orthopedics Qilu Hospital Shandong University Jinan Shandong China; ^2^ Department of Orthopedics, Qilu Hospital (Qingdao) Shandong University Qingdao Shandong China

**Keywords:** bioactive hydrogel, bone defect repair, bone marrow mesenchymal stem cells, bone regeneration, vascular regeneration

## Abstract

Critical‐sized bone defects (CSD) remain a major clinical challenge due to three interrelated barriers: inadequate mechanical support, insufficient osteogenic induction, and impaired angiogenesis, all of which hinder effective regeneration. To tackle these, we developed a dual‐network bioactive scaffold, ermd bFGF@CB‐gel, based on a chondroitin sulfate methacryloyl/bacterial cellulose gel (CB‐gel) which synergistically combines three key properties: i) a photocurable biomimetic mineralized scaffold (CB‐gel) for in situ bone repair with mechanical support and a bone‐ECM‐mimicking microenvironment for delivering bone marrow mesenchymal stem cells (BMSCs); ii) a bio‐nano carrier (BC) for sustained release of bFGF which enhances the adhesion and proliferation via EGFL/Itga2b pathway, strengthens osteogenic differentiation and mineralization by activating the COMP/PI3K/AKT pathway of rat BMSCs; iii) bFGF released by the dual‐network promotes migration and angiogenesis of microvascular endothelial cells by combining FGFR to activate the PI3K/AKT/eNOS pathway. In a rat CSD model, the bFGF@CB‐gel achieved a statistically significant increase in new bone volume, as quantified by micro‐CT, and enhanced vascular density, evaluated via immunohistochemical staining. These findings highlight the potential of bFGF@CB‐gel as an effective local delivery system of BMSCs via linking biomechanics, molecular signaling, and cellular activity, which moves beyond simplistic function stacking to a rational, synergistic design for bone regeneration in CSD, addressing key challenges in reconstructive surgery.

## Graphical abstract

A dual‐network bFGF@CB‐gel integrates: i) ChSMA scaffold (mechanical support/BMSCs delivery); ii) BC carrier (sustained bFGF release via EGFL/Itga2b and COMP/PI3K/AKT for adhesion/osteogenesis); iii) bFGF/PI3K/AKT/eNOS for angiogenesis. This rational, synergistic design addresses CSD bone regeneration challenges.

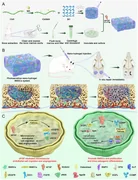

## 1 Introduction

Critical‐sized bone defects (CSD) refer to bone defects induced by events such as trauma, infection, tumor removal, or nonunion of osteoporotic fractures that surpass the self‐healing potential of bone [1, 2, 3, 4]. Although bone offers natural regeneration potential, contemporary autologous transplantation poses challenges such as secondary injury and lengthy surgical time [5, 6], whereas allogeneic transplantation is limited by donor scarcity and dangers of immunological rejection. Therefore, the regeneration of CSD remains a critical therapeutic problem, urgently necessitating the development of effective and safe interventional procedures.

Compelling evidence demonstrates that bone substitute materials are crucial for attaining functional restoration and complete osseous repair. The production and repair of bone tissue constitute a highly coordinated dynamic process involving intricate interactions among different cell types and tissue components, ultimately generating neo‐mineralized tissues containing vascular endothelium, autonomic nerves, and sensory nerves. This process is predominantly driven by osteoblasts [7, 8], while blood arteries operate as structural scaffolds during bone creation, carrying critical components, such as mineral salts, growth hormones, and osteogenic cells‐to the osteoblastic milieu, thereby maintaining bone homeostasis [9]. Cellular aging, unstable differentiation, and poor angiogenesis affect bone remodeling, with bone resorption surpassing production, hindering regeneration at defect locations [10, 11].

Rebuilding a pro‐regenerative milieu is pivotal for bone repair, in which angiogenesis supports osteogenesis by supplying oxygen/nutrients and orchestrating vascular‐bone crosstalk. Bone marrow mesenchymal stem cells (BMSCs), first described by Friedenstein in 1976 [12], exhibit robust osteogenic potential and provide hematopoietic support [12, 13, 14, 15, 16], making them attractive candidates for cell‐based regeneration. Basic fibroblast growth factor (bFGF) further augments repair by sustaining BMSCs proliferation and osteogenic commitment [17], while promoting endothelial activation and neovessel formation [18, 19, 20, 21]. Accordingly, biomaterial‐assisted strategies‐particularly hydrogel‐based platforms‐have pursued the combined delivery of BMSCs and bFGF to reconstruct a pro‐regenerative niche that couples osteogenesis with angiogenesis [22, 23, 24, 25, 26, 27]. For instance, bFGF‐transfected BMSCs combined with mineral scaffolds improved mandibular regeneration [28], and bFGF has been incorporated into MSC constructs (e.g., spheroids) to potentiate fracture healing through localized trophic cues [29].

However, clinical translation remains hindered by poor BMSCs survival in the hostile post‐implantation milieu [30, 31, 32] and by the instability/high diffusibility of bFGF with potential dose‐dependent inhibitory effects [33]. Although hydrogels are widely used for co‐delivery, many systems are mechanically insufficient and fail to sustain labile growth‐factor presentation in critical‐sized defects [34, 35]. Consequently, affinity ligands (e.g., heparin/heparan sulfate), micro/nanocarriers, or covalent immobilization are often introduced to curb bFGF burst release, at the cost of added complexity and potentially compromised injectability or cytocompatibility‐even in dual‐/interpenetrating‐network designs [36, 37]. Together, these limitations motivate injectable platforms that couple mechanical reinforcement with ECM‐like architecture and intrinsic, carrier‐free stabilization of bFGF over a meaningful regenerative window, while enabling mechanistic dissection of bFGF‐driven osteogenesis‐angiogenesis coupling.

Glycosaminoglycan (GAG)‐ and cellulose‐based matrices offer complementary advantages for bone repair. Chondroitin sulfate regulates cellular behavior and extracellular matrix production [38, 39, 40, 41, 42, 43], whereas bacterial cellulose (BC) provides a nanofibrous, ECM‐mimetic architecture with high strength, biocompatibility, and water retention [44, 45, 46, 47, 48]. To integrate GAG biofunctionality with fibrous reinforcement, Chondroitin sulfate has been combined with BC to generate more bioactive scaffolds. For example, CS‐incorporated macroporous BC enhances cell adhesion/proliferation and mineral deposition [49], and Chondroitin sulfate‐assisted biosynthesis/mineralization promotes calcium phosphate deposition, suggesting improved osteoconductivity [50]. BC has also been integrated with osteoinductive components (e.g., collagen or calcium phosphate/hydroxyapatite) to enhance neovascularization and bone formation in vivo [51]. However, most Chondroitin sulfate/BC constructs are non‐photocrosslinkable scaffolds or surface coatings, limiting minimally invasive delivery and in situ conformal filling. In contrast, Chondroitin sulfate methacryloyl (ChSMA; hereafter abbreviated as CS) enables rapid, mild photocrosslinking and injectable processing [52, 53]. Thus, an injectable, photocurable ChSMA hydrogel reinforced by BC that provides intrinsic GAG‐mediated bioactivity while stabilizing bFGF presentation remains insufficiently explored.

**FIGURE 1:**
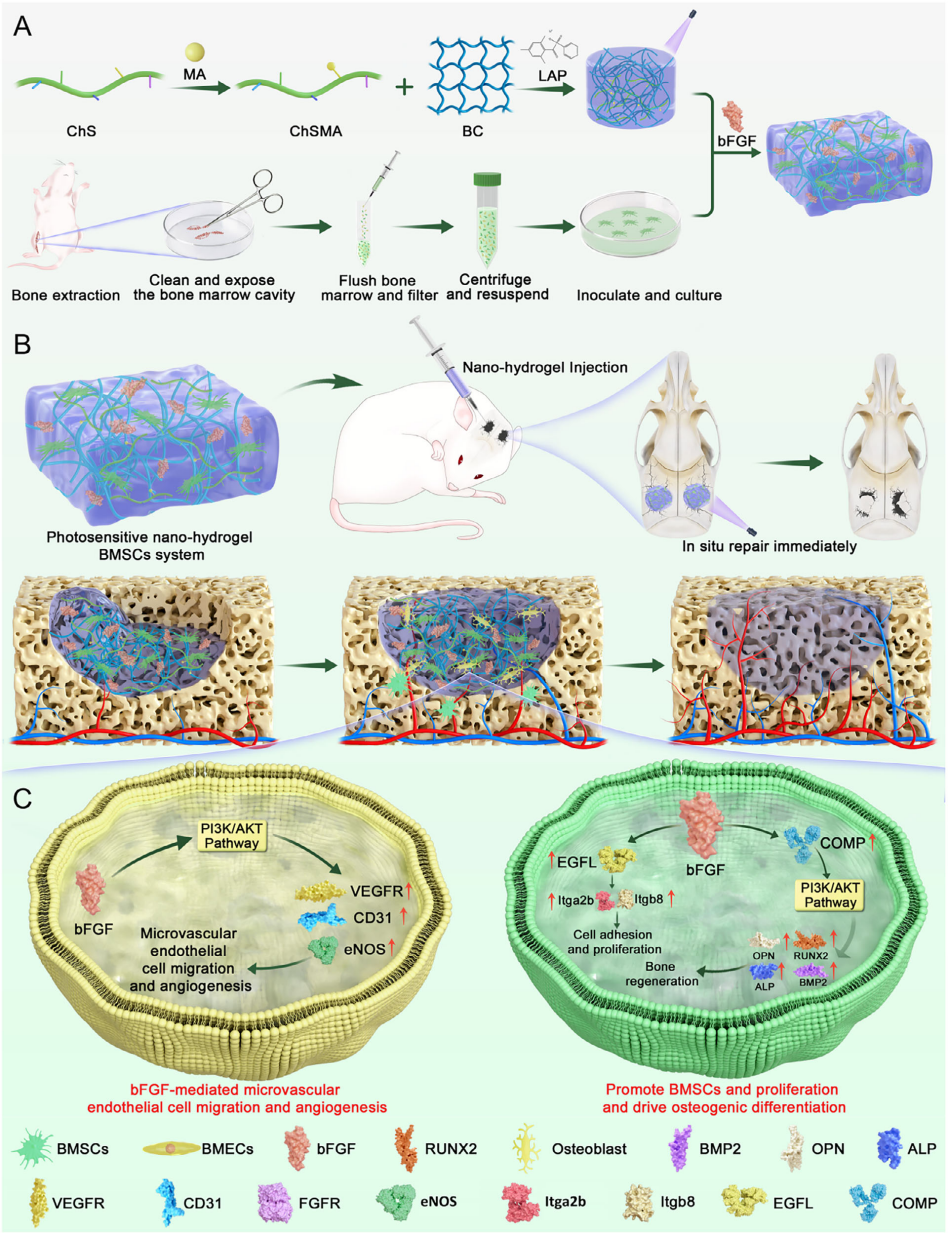
Schematic illustration of the photosensitive nano‐hydrogel (bFGF@CB‐gel) system for BMSCs delivery to facilitate bone regeneration. (A). Synthesis of bFGF@CB‐gel via photochemical crosslinking and extraction and loading of BMSCs. (B). Transplantation procedure of the BMSCs‐encapsulated bFGF@CB‐gel hydrogel system. (C). Mechanisms of osseous defect repair.

Here, we developed an injectable, in situ photocurable ChSMA/BC dual‐network hydrogel (ChSMA/BC gel; hereafter abbreviated as CB‐gel), comprising a covalently crosslinked ChSMA network interpenetrated with a BC nanofiber‐derived physical network (Figure 1). BC serves as both a mechanical reinforcing phase and an intrinsic reservoir that stabilizes and sustains bFGF presentation, enabling one‐step co‐encapsulation of BMSCs and bFGF without additional carriers. Benefiting from the nanofibrous architecture, CB‐gel provides high‐surface‐area topographical cues that support MSC attachment and proliferation. Importantly, we further interrogate the pro‐angiogenic and pro‐osteogenic contributions of bFGF at key regenerative stages in vivo, linking early vascular responses to later bone formation. Consequently, bFGF@CB‐gel promotes osteogenesis and angiogenesis in vitro and markedly enhances bone regeneration and neovascularization in a rat calvarial CSD model. Mechanistically, transcriptomic profiling together with protein‐level validation suggests the involvement of PI3K/AKT signaling and ECM‐related programs. Specifically, our data indicate that EGFL/Itga2b is associated with enhanced BMSCs adhesion and proliferation, COMP/PI3K/AKT is linked to strengthened osteogenic differentiation and mineralization in rat BMSCs, and PI3K/AKT/eNOS is implicated in microvascular endothelial cell migration and angiogenesis, collectively supporting a coupled osteogenesis–angiogenesis mechanism within the bFGF@CB‐gel–mediated BMSCs delivery system. To the best of our knowledge, this is the first report suggesting that these coordinated adhesion‐, osteogenesis‐, and angiogenesis‐related pathways may be concurrently engaged within a BC‐reinforced photocurable GAG dual‐network hydrogel microenvironment that serves as a local delivery matrix for BMSCs.

## 2 Results

### 2.1 Characterization of Hydrogel Systems

**FIGURE 2:**
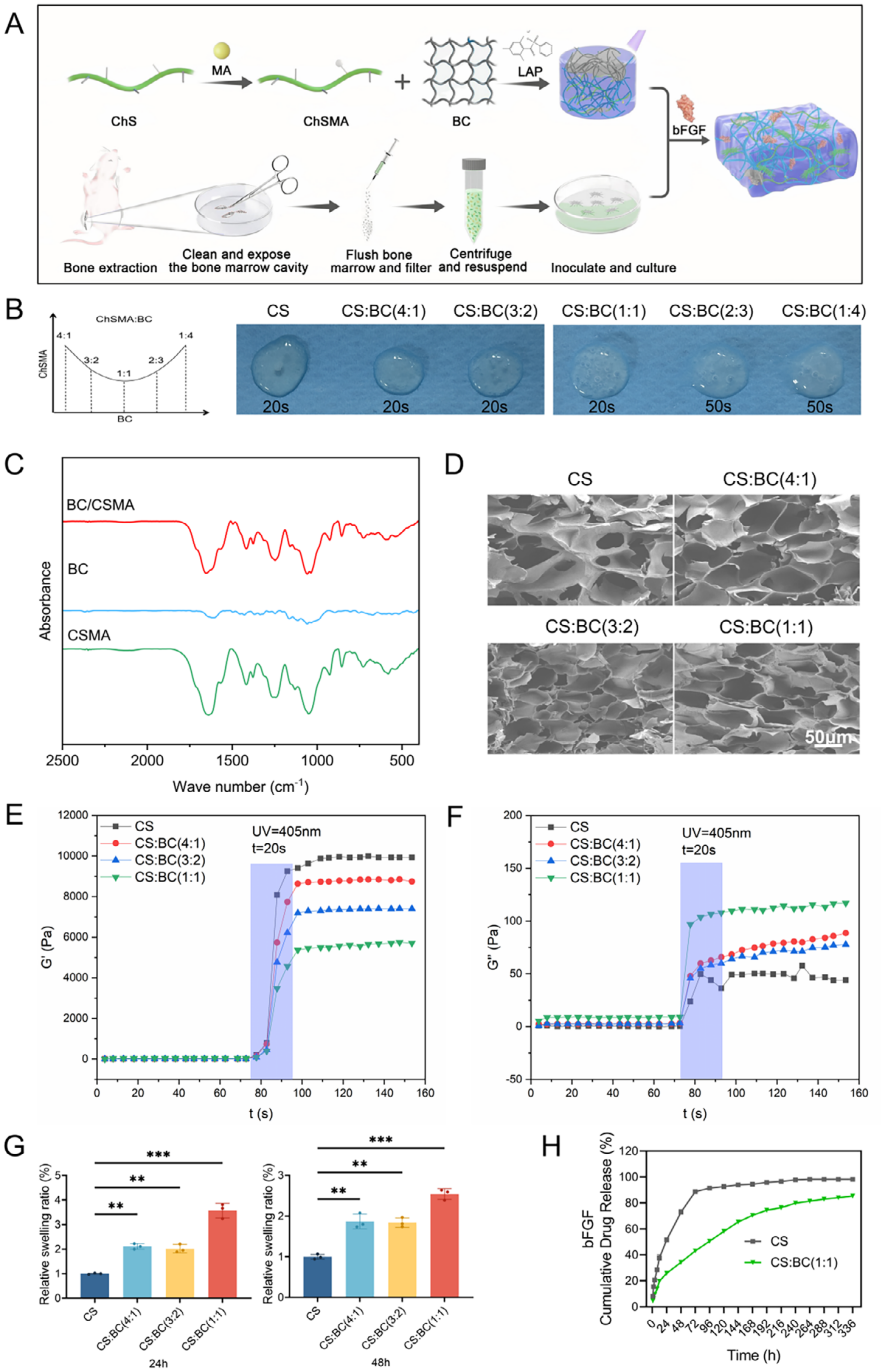
Characterisation of hydrogel. (A). Schematic representation of hydrogel synthesis mediated by photocrosslinking. (B). Exploration of the CS and BC ratio and macroscopic pictures at different ratios. (C). FTIR analysis confirmed hydrogen‐bond crosslinking between BC and ChSMA. (D). Cross‐section image of hydrogel (SEM). (E,F). The Dynamic Mechanical Analysis of CB‐gel(storage modulus (G') and loss modulus (G'')). (G). Time‐dependent swelling kinetics of hydrogels at 24 and 48 h. (H). Analysis of bFGF Cumulative Drug Release from CB‐gel(CS:BC(1:1)). The data are presented as mean ± SD, *n* = 3. ^*^ *p* < 0.05, ^**^ *p* < 0.01 and ^***^ *p* < 0.001.

In this study, photocurable hydrogels with varying ChSMA‐to‐BC ratios were prepared (Figure 2A). Macroscopic observations (Figure 2B) showed that reducing the ChSMA volume fraction from 100% to 50% enabled all formulations to gel stably within 20 s under 405‐nm irradiation, whereas the CS:BC = 2:3 and 1:4 groups required ∼50 s for complete gelation. Among the rapidly gelling formulations, the ChSMA/BC hydrogel (CB‐gel; CS:BC = 1:1) formed a homogeneous and transparent 3D network. FTIR spectra indicated strengthened hydrogen‐bonding interactions between BC and ChSMA, supporting their intermolecular association in the composite formulation (Figure 2C). Consistently, SEM imaging showed well‐organized porous architectures in the ∼20 s gelled formulations (Figure 2D), while the ∼50 s groups exhibited disorganized pores, heterogeneous pore sizes, and reduced interconnectivity (Figure S1A).

To prioritize an optimal formulation for subsequent biological evaluation, we next compared mechanical and physicochemical properties. Under 405‐nm irradiation (20 s), DMA showed that the CB‐gel (CS:BC = 1:1) achieved a storage modulus (G′) of ∼6000 Pa (Figure 2E), and all formulations displayed elastic‐dominated behavior (G′ > G″) (Figure 2F). The CB‐gel also exhibited favorable swelling stability (Figure 2G). Collectively, these FTIR, microstructural, viscoelastic, and swelling results supported selecting the CB‐gel (CS:BC = 1:1) for further cell and release studies.

Subsequently, to assess cellular adhesion, BMSCs were seeded onto the CS‐gel (without BC) and BC‐reinforced CB‐gel for 24 h. Phalloidin staining of F‐actin showed that, compared with the CS‐gel, cells on the CB‐gel displayed more pronounced spreading with multidirectional protrusions and abundant filopodia/lamellipodia, indicating improved cytoskeletal organization and adhesion (Figure S1E). Moreover, a CCK‐8 assay further showed higher cell proliferation in the CB‐gel than in the CS‐gel and Control groups (Figure S1B). Finally, an in vitro cumulative release assay demonstrated a slower and more prolonged bFGF release from the CB‐gel (Figure 2H). The cumulative release profiles were further fitted with three classical kinetic models (Figure S1C,D).

### 2.2 bFGF@CB‐gel Displays Good Biocompatibility

In vitro, BMSCs were combined with composite hydrogels of different CS/BC concentration ratios and co‐cultured. The cytocompatibility was tested using a live‐dead cell double staining experiment (Figure S2A). After 7 days of incubation, all experimental groups displayed good cell viability (>90%), with the CB‐gel (CS:BC = 1:1) showing the most homogenous cell distribution and minimal dead cell presence (red fluorescence) (<5%). Quantitative analysis (Figure S2B) demonstrated that cell survival in the CB‐gel (CS:BC = 1:1) group was superior to that in the CS‐gel group, demonstrating that BC significantly boosted material cytocompatibility. These findings validated the high in vitro biocompatibility of the CB‐gel (CS:BC = 1:1) composite hydrogel and provide preliminary screening data for appropriate bFGF concentration.

This study collected rat heart, liver, spleen, lung, and kidney tissues at 12 weeks for hematoxylin–eosin (H&E) staining examination and blood samples for liver and kidney function tests. H&E staining of major organs (heart, liver, spleen, lung, and kidney) indicated intact tissue architecture in the experimental group, with no pathological alterations seen (Figure S4A). Hematological analysis demonstrated that liver function indicators (ALT, AST, ALP) and renal function indicators (UA, BUN, CR) of rats in all groups were within normal physiological ranges (Figure S4B). These in vivo findings further demonstrate the favorable systemic biocompatibility of the material.

### 2.3 bFGF@CB‐gel Promotes In Vitro Angiogenic Responses

Prior to evaluating the in vitro osteogenic and angiogenic performance of the hydrogel, we first investigated the effects of different concentrations of bFGF on osteogenic differentiation to determine the optimal bFGF concentration. Imunofluorescence staining for RUNX2 demonstrated that all experimental groups treated with 50, 100, and 200 ng/mL bFGF exhibited significantly elevated RUNX2 protein expression compared to the control group. Notably, the group treated with 100 ng/mL bFGF had the highest fluorescence intensity, a finding corroborated by quantitative analysis, which showed considerably enhanced RUNX2 expression in this group relative to the control (Figure S3A,B). Additionally, RT‐qPCR examination of major osteogenic markers revealed that the mRNA expression levels of Alpl, Ocn, Opn, and Runx2 (Figure S3C) were considerably increased in the 100 ng/mL bFGF‐treated group. Additionally, cell growth kinetics measured by CCK‐8 assay revealed that the bFGF‐100 group exhibited considerably greater absorbance values than the other groups at 4 and 7 days of culture (Figure S3D). These findings were consistent with the protein‐level results. Collectively, these data indicate that 100 ng/mL is the optimal bFGF concentration for promoting osteogenic differentiation and was therefore selected for subsequent experiments.

**FIGURE 3:**
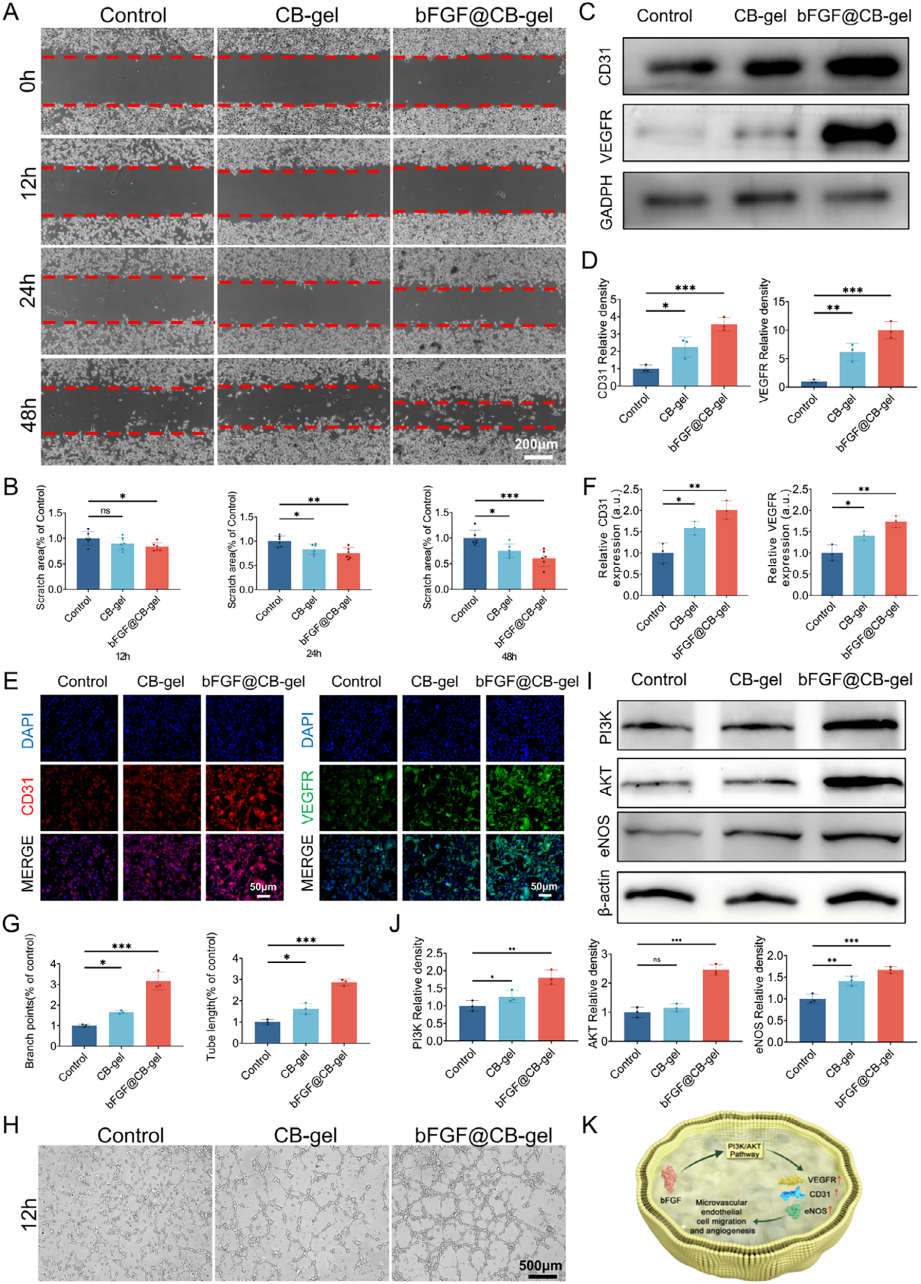
bFGF@CB‐gel stimulates angiogenesis in vitro. (A). Migration assay of BMECs. (B). Quantification of migration assay at 12, 24, and 48 h. (C,D). Western blot and its quantification of CD31 and VEGFR in BMECs after co‐culture. (E,F). Immunofluorescence staining and quantification of CD31 and VEGFR in BMECs after co‐culture. (G,H). Representative images of Matrigel tube formation by BMECs after co‐culture(H) and the quantitative analysis(G) of tube formation, including total tube length and branch points in BMECs post co‐culture. (I,J). Western blot and its quantification of PI3K, AKT, and eNOS in BMECs post‐co‐culture. (K). Schematic illustration of the mechanism underlying bFGF‐promoted angiogenesis. The data are presented as mean ± SD, *n* = 3. ^*^p < 0.05, ^**^ *p* < 0.01, and ^***^p < 0.001.

Angiogenesis, a pivotal process in bone repair that supplies essential substrates for osteoblasts and secretes osteogenic factors to markedly enhance bone formation, is further amplified by bFGF, which not only drives the osteogenic activity of BMSCs but also modulates endothelial cell function to promote neovascularization, thereby achieving synergistic effects between osteogenesis and angiogenesis. Therefore, confirming the pro‐angiogenic capacity of bFGF is vital for comprehending its mechanism in promoting bone regeneration.In this study, we thoroughly examined the pro‐angiogenic capability of CB‐gel composite hydrogels loaded with bFGF through complementary functional assays and molecular analyses. Transwell migration tests were performed to evaluate the migration behavior of rat brain microvascular endothelial cells (BMECs) at 12, 24, and 48 h (Figure 3A). Compared with the control group, the bFGF@CB‐gel group showed a significant increase in migrating cells at 12 h, peaking at 24 h, with sustained high migration activity at 48 h. Quantitative analysis (Figure 3B) further confirmed a time‐dependent increase in the migration rate in the bFGF@CB‐gel group, indicating a robust chemotactic response. Western blot analysis revealed that the bFGF@CB‐gel group exhibited higher protein levels of VEGFR and CD31 compared with the control group (Figure 3C,D). Consistently, immunofluorescence staining and quantitative analyses further confirmed significant upregulation of these angiogenesis‐related markers (CD31 and VEGFR) in the bFGF@CB‐gel group (Figure 3E,F). Moreover, we supplemented a Matrigel‐based endothelial tube formation assay, a widely used in vitro angiogenesis assay, to assess the ability of BMECs to form capillary‐like networks. Representative images showed more extensive and interconnected tube‐like structures in the bFGF@CB‐gel group (Figure 3H). Quantification using the ImageJ Angiogenesis Analyzer demonstrated significantly increased total tube length, as well as higher numbers of junctions and meshes in the bFGF@CB‐gel group (Figure 3G). To explore the mechanistic basis of enhanced angiogenesis by bFGF@CB‐gel, we focused on the PI3K/AKT signaling pathway, which is recognized as pivotal in bFGF‐mediated angiogenic responses. Western blot analysis was performed to assess PI3K/AKT expression, alongside detection of endothelial nitric oxide synthase (eNOS), a downstream effector implicated in endothelial function (Figure 3I,J). Mechanistically, we hypothesized that bFGF binding to fibroblast growth factor receptors (FGFRs) engages PI3K/AKT signaling, which subsequently induces eNOS activation in endothelial cells; these molecular alterations collectively underpin the augmented migratory and pro‐angiogenic phenotype observed in the bFGF@CB‐gel group (Figure 3K). Collectively, these functional and molecular results demonstrate that CB‐gel composite hydrogels loaded with bFGF exert pronounced pro‐angiogenic effects by PI3K/AKT/eNOS signaling in vitro.

### 2.4 bFGF@CB‐gel Enhances In Vitro Osteogenic Commitment and Mineralization

Osteogenic differentiation is indispensable for effective bone repair, as it directly governs new bone matrix deposition and mineralization. Therefore, we next investigated the in vitro osteoinductive capacity of CB‐gel composite hydrogels loaded with bFGF.

**FIGURE 4:**
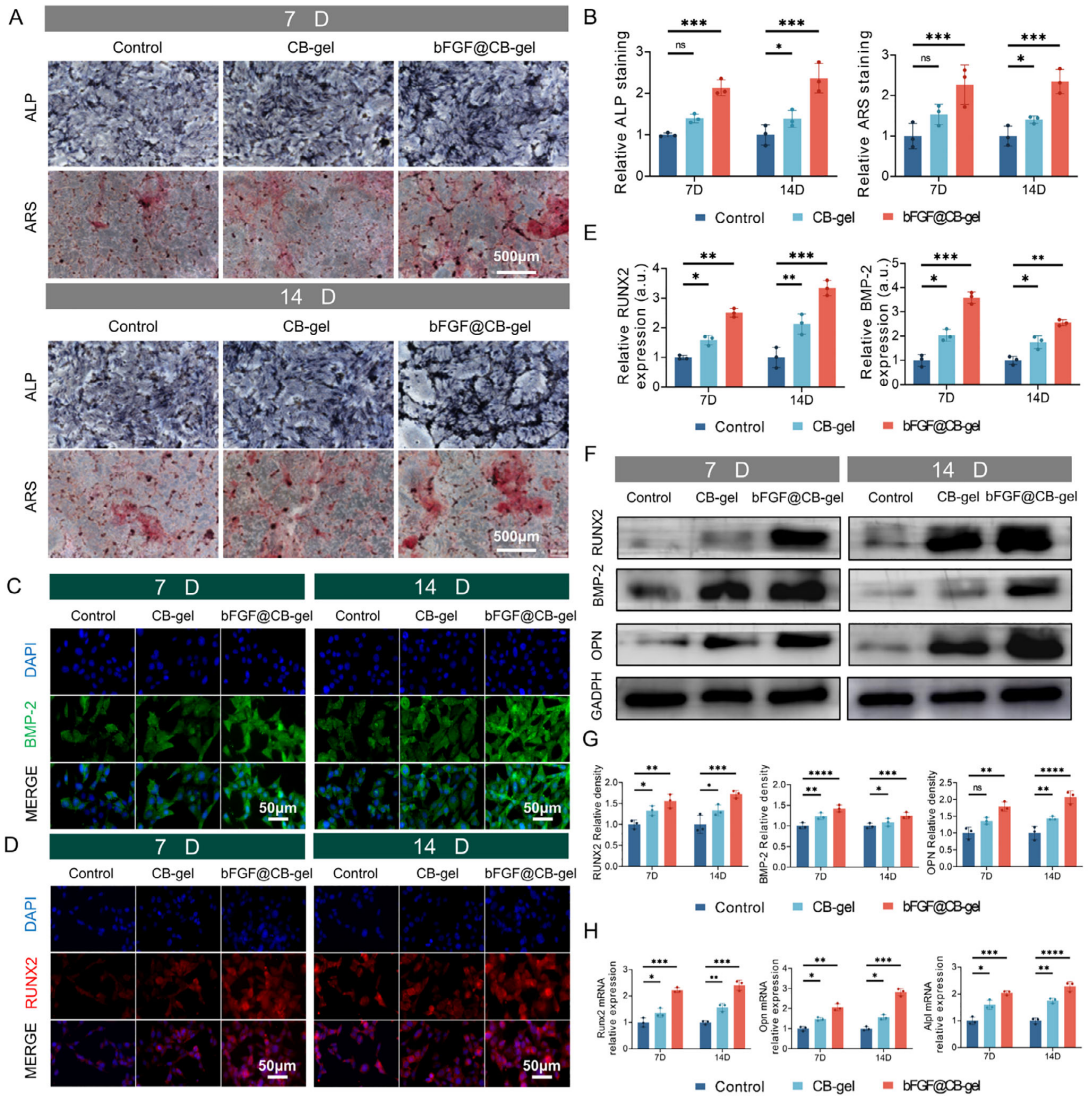
bFGF@CB‐gel promotes osteogenic differentiation. (A). ALP and ARS staining of BMSCs co‐cultured at 7‐ and 14 D. (B). Quantitative analysis of ALP and ARS staining. (C,D). Immunofluorescence(IF) staining of BMSCs after co‐cultured at days 7 and 14(BMP‐2, green; RUNX2, red). (E). Quantitative analysis of IF staining at days 7 and 14. (F). Western blot analysis(WB) of RUNX2, BMP‐2, and OPN in BMSCs after co‐cultured at days 7 and 14. (G). Quantitative analysis of WB at days 7 and 14. H). qPCR analysis of *Runx2*, *Alpl*, and *Opn* expression in BMSCs after co‐cultured at days 7 and 14. The data are presented as mean ± SD, *n* = 3. ^*^ *p <* 0.05, ^**^ *p <* 0.01 and ^***^ *p <* 0.001.

In vitro osteogenic differentiation assays demonstrated that CB‐gel loaded with bFGF exerted a considerable osteoinductive effect. ALP and ARS staining (Figure 4A) and quantitative analysis (Figure 4B) demonstrated that ALP expression was considerably raised in the bFGF@CB‐gel group compared to the control group on day 7 and subsequently increased on day 14. Mineralized nodule formation increased dramatically with longer incubation time, with markedly more calcium deposition detected at 14 days than at 7 days. Immunofluorescence staining (Figure 4C,D) and quantitative analysis (Figure 4E) demonstrated that BMP‐2 and RUNX2 expression levels in the bFGF@CB‐gel group were considerably greater than those in the control and CB‐gel groups and further increased after 14 days compared to 7 days. Western blot analysis (Figure 4F,G) showed higher expression of key osteogenic regulatory proteins in the bFGF@CB‐gel group than in the control group, with distinct temporal expression patterns: RUNX2 was significantly upregulated on day 7 and remained elevated through day 14; BMP‐2 exhibited an early rapid increase before returning to baseline; and OPN, a late marker, showed further increased expression on day 14 compared to day 7. These findings corroborate the osteogenic capability of CB‐gel composite hydrogels loaded with bFGF and reveal temporal regulatory shifts during osteogenic development. RT‐qPCR analysis (Figure 4H) supported the protein‐level results, with *Runx2* and *Alpl* gene expression significantly raised at 7 days and *Opn* gene expression further enhanced at 14 days. Collectively, our data demonstrate the in vitro osteogenic induction capacity of CB‐gel composite hydrogels loaded with bFGF and reveal the temporal expression dynamics of early (BMP‐2/RUNX2/ALP) and late (OPN/mineralization) markers during osteogenic differentiation.

### 2.5 bFGF@CB‐gel Promotes Osteogenesis via the COMP/PI3K/AKT Signaling Pathway

**FIGURE 5:**
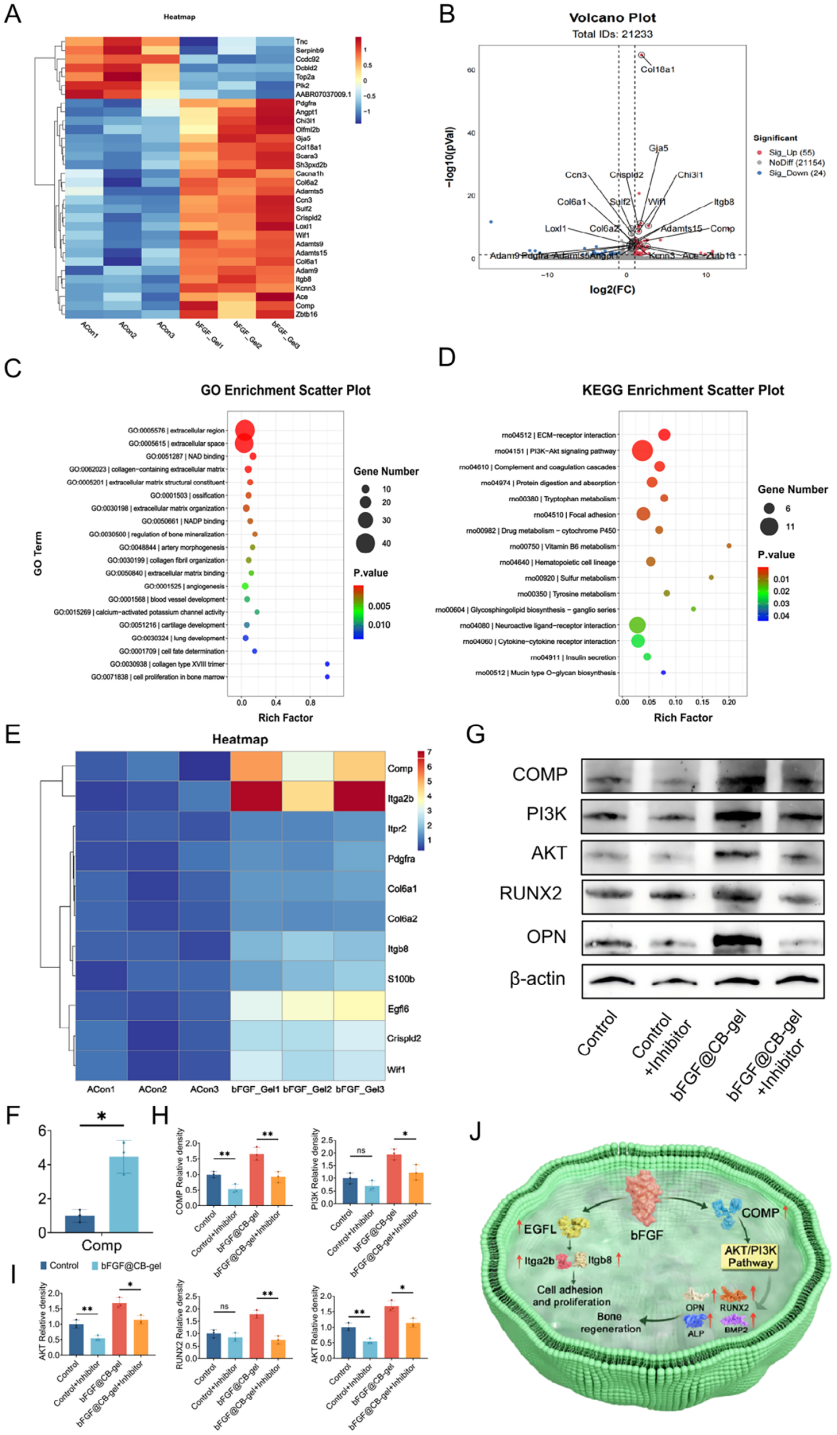
RNA‐seq validation of the bone repair mechanism facilitated by the bFGF@CB‐gel hydrogel system. (A). Heatmap of upregulated genes. (B). Volcano plot identifying DEGs. (C,D). KEGG pathways dominated by PI3K/AKT signaling. (E,F) COMP was the representative upstream ECM cue and verified its upregulation by transcriptomic analysis in the PI3K/AKT pathway. (G–I) Western blot and quantitative analysis confirming pathway activation. (J). Schematic Diagram of Osteogenic Mechanism.The data are presented as mean ± SD, n = 3. *p < 0.05, **p < 0.01 and ***p < 0.001.

In this study, the osteogenic mechanism of the bFGF@CB‐gel composite was analyzed by RNA‐seq (Figure 5). Heat map analysis (Figure 5A) demonstrated that the experimental group markedly upregulated extracellular matrix‐related genes (e.g., *Col6a1/2* and *Adamts5/9/15*) and pro‐angiogenic factors (e.g., *Angpt1* and *Pdgfra*). Volcano plots (Figure 5B) identified 79 differentially expressed genes, and GO analyses (Figure S5A,B) suggested that extracellular matrix remodeling (GO:0030199) and myeloid proliferation (GO:0071838) were among the fundamental biological processes. KEGG enrichment analysis (Figure 5C,D) identified the PI3K/AKT pathway as a major hub linking ECM‐receptor interaction with metabolic reprogramming, and the activation of this pathway was further supported by Western blotting (Figure S5C,D). To further connect transcriptomic signals to functional regulation, we selected cartilage oligomeric matrix protein (COMP) as a representative upstream ECM cue and verified its upregulation by transcriptomic analysis in the PI3K/AKT pathway (Figure 5E,F). Combined with literature evidence, COMP was identified as an upstream regulator of the PI3K/AKT pathway. Following treatment with a PI3K/AKT pathway inhibitor, WB detection elucidated their functional relationship, demonstrating that bFGF promotes osteogenic mineralization via the COMP/PI3K/AKT pathway (Figure 5G–I). Considering previous findings (Figure S1E) that indicated enhanced proliferation, we analyzed sequencing data and observed high expression of proliferation‐adhesion genes (e.g., *Itgb8 and Itga2b*), thereby enhancing cell proliferation and ECM‐binding capacity. We further validated key nodes of this adhesion pathway, including Itgb8, Itga2b, and EGFL6, by Western blotting (Figure S1F,G). Collectively, these results corroborate the classic PI3K/AKT‐mediated osteogenic program and provide direct inhibitory evidence supporting a bFGF‐driven cell‐adhesion/proliferation mechanism within the hydrogel microenvironment that contributes to enhanced bone regeneration. Finally, the underlying mechanism was summarized in a schematic diagram (Figure 5J).

### 2.6 bFGF@CB‐gel–Mediated BMSC Delivery Accelerates Vascularized Bone Regeneration In Vivo

**FIGURE 6:**
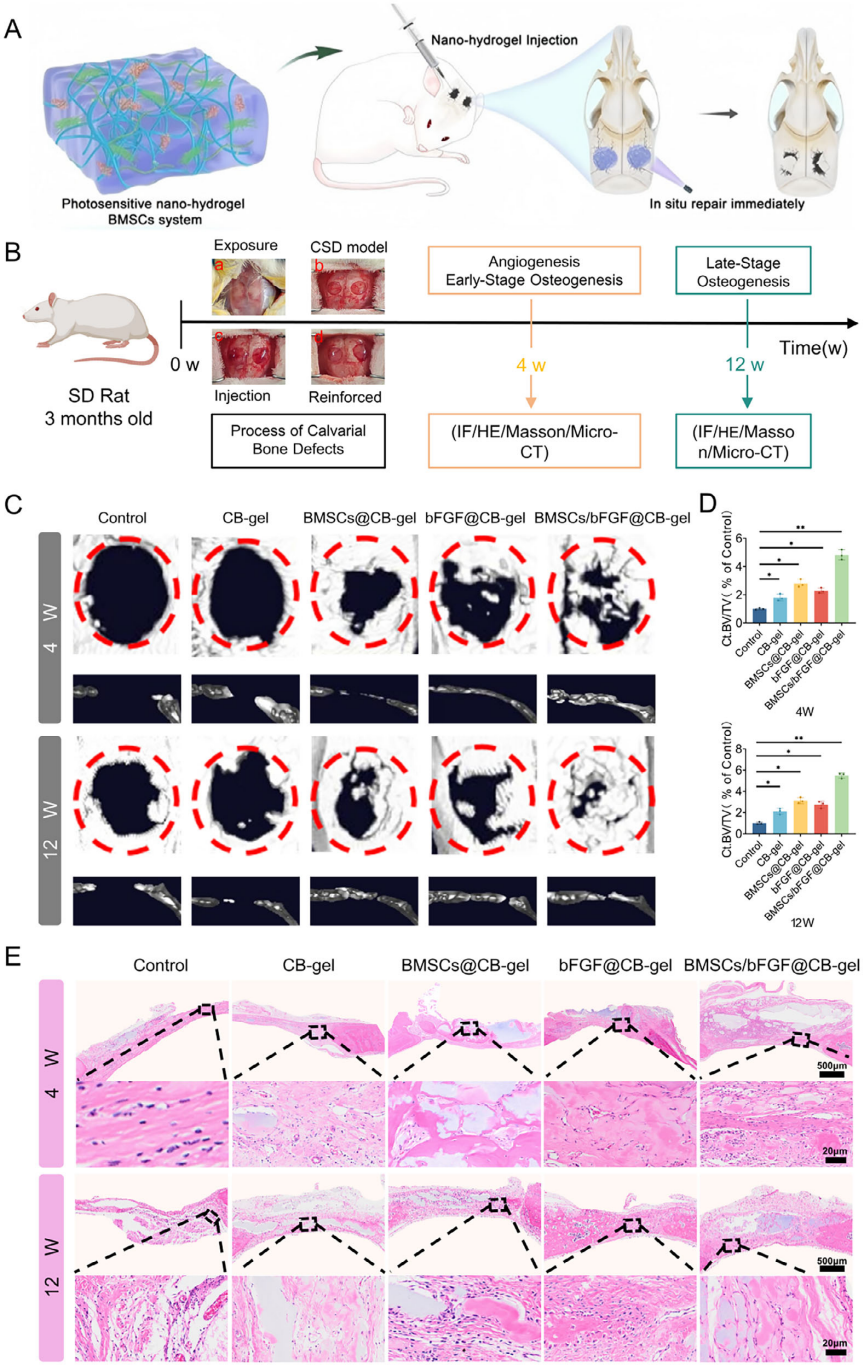
bFGF@CB‐gel–mediated BMSCs delivery promotes new bone regeneration in vivo. (A). Schematic representation of cranial defect modeling and hydrogel implantation. (B). Schematic diagram of animal experimental design. (C). Representative Micro‐CT images at 4 and 12 weeks after surgery in the calvarial defect model. (D). Quantitative analysis of BV/TV at 4 and 12 weeks after surgery in cranial defect. (E). H&E staining at 4 and 12 weeks after surgery in cranial defects, showing new bone mainly at defect margins with residual soft tissue in the central region. The data are presented as mean ± SD, *n* = 3. ^*^ *p <* 0.05, ^**^ *p* < 0.01 and ^***^ *p* < 0.001.

In vitro experiments showed that the CB‐gel composite hydrogel loaded with bFGF exhibited excellent cytocompatibility as well as pronounced angiogenic and osteoinductive activities. To further evaluate the potential of the hydrogel as a BMSCs delivery platform to promote angiogenesis and osteogenesis, an in vivo rat calvarial CSD model was developed. Bilateral cranial defects, each 5 mm in diameter, were surgically created and then implanted with different hydrogel formulations, followed by in situ cross‐linking under 405 nm ultraviolet light (Figure 6A). The detailed design of the animal test protocol is shown in Figure 6B.

#### 2.6.1 bFGF@CB‐gel–Mediated BMSC Delivery Upregulates Angiogenesis‐Associated Proteins In Vivo

**FIGURE 7:**
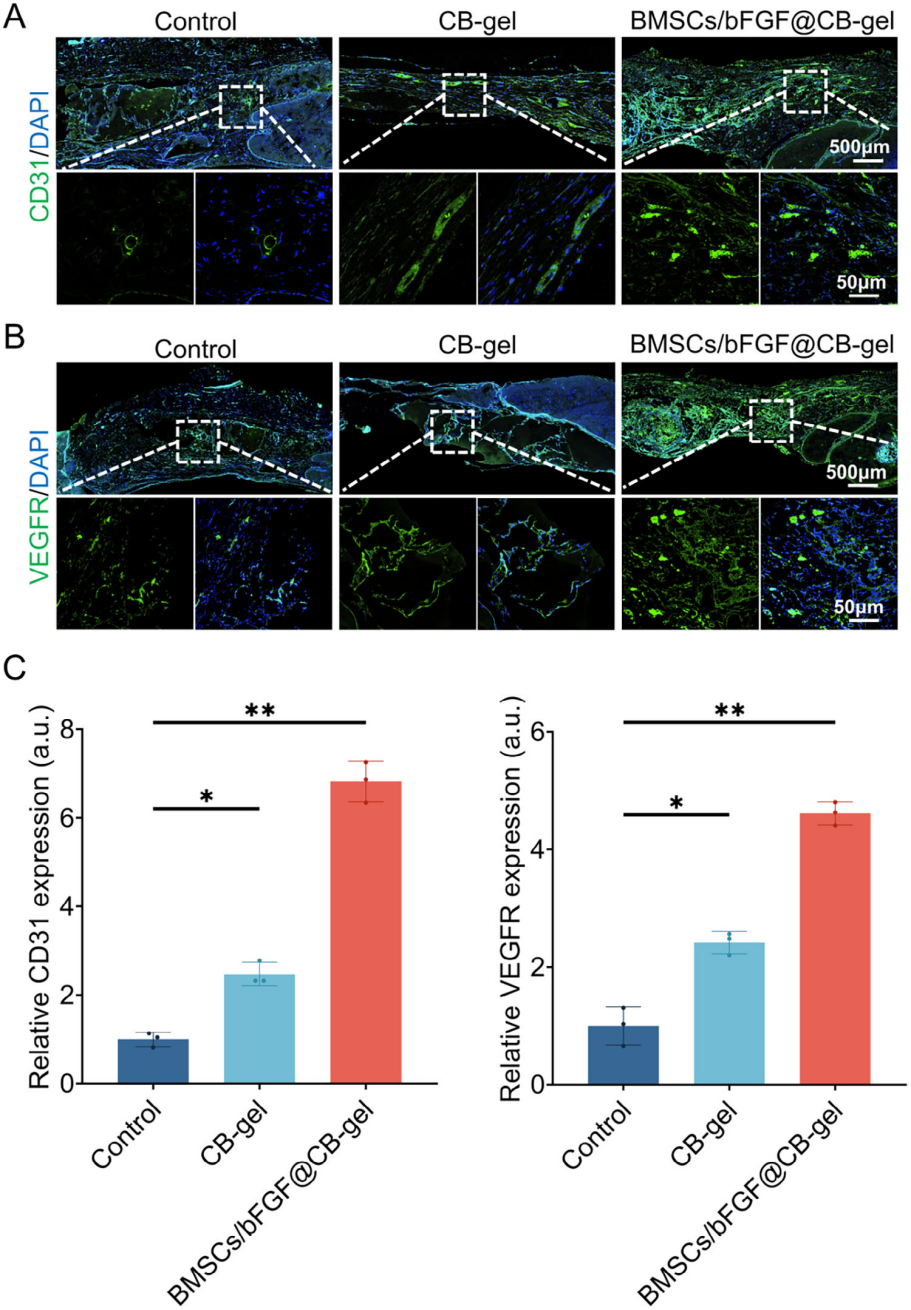
bFGF@CB‐gel–mediated BMSCs delivery stimulates the production of angiogenesis‐related proteins in vivo. (A,B). Immunofluorescence staining of CD31(A) and VEGFR(B) at 4 weeks after surgery in cranial defect. (C). Quantification of CD31 and VEGFR immunofluorescence at 4 weeks after surgery in cranial defect. The data are presented as mean ± SD, *n* = 3. ^*^ *p <* 0.05, ^**^ *p <* 0.01 and ^***^ *p <* 0.001.

At 4 weeks post‐operation, harvested specimens were systematically analyzed for angiogenesis‐related proteins and osteogenic markers. Immunofluorescence staining revealed significantly higher expression of the endothelial markers CD31 and VEGFR in the bFGF@CB‐gel–mediated BMSCs delivery group (BMSCs/bFGF@CB‐gel) than in the other groups (Figure 7). Importantly, we further conducted immunofluorescence staining of HIF‐1α and endomucin (EMCN), which showed an overall enhanced pro‐angiogenic microenvironment and increased formation of EMCN‐positive microvessels in the BMSCs/bFGF@CB‐gel group (Figure S6). These in vivo observations are consistent with our in vitro findings, where angiogenesis‐related proteins (including HIF‐1α‐associated signaling and endothelial markers) were upregulated at the protein level.

Collectively, these results support a close coupling between early osteogenesis and vascular regeneration during bone healing: newly formed vessels not only improve local nutrient and oxygen delivery but may also modulate osteogenic differentiation through paracrine signaling. Therefore, angiogenesis appears to play not merely a supportive role, but a potentially regulatory role in orchestrating the early stage of bone regeneration.

#### 2.6.2 bFGF@CB‐gel–Mediated BMSC Delivery Augments New Bone Formation and Osteogenic Marker Expression In Vivo

Meanwhile, the hydrogel matrix appeared to be substantially degraded in vivo by week 4 based on gross observation and histology (Figure S7), suggesting completion of its primary carrier function during the early healing phase; however, the residual gel volume/mass was not quantitatively measured in this work. Micro‐computed tomography (Micro‐CT) reconstruction (Figure 6C) revealed increased mineralized tissue within the defect in all groups, and the BMSCs/bFGF@CB‐gel group showed the most pronounced bone ingrowth, mainly extending from the defect margins. Quantitative analysis of the bone volume fraction (BV/TV) further confirmed the enhanced osteogenic effect of the BMSCs/bFGF@CB‐gel (Figure 6D). Consistently, H&E staining (Figure 6E) showed newly formed bone at the defect edges and the hydrogel‐host interface, whereas the central region still contained non‐mineralized soft tissue, suggesting that defect bridging was ongoing at this time point.

**FIGURE 8:**
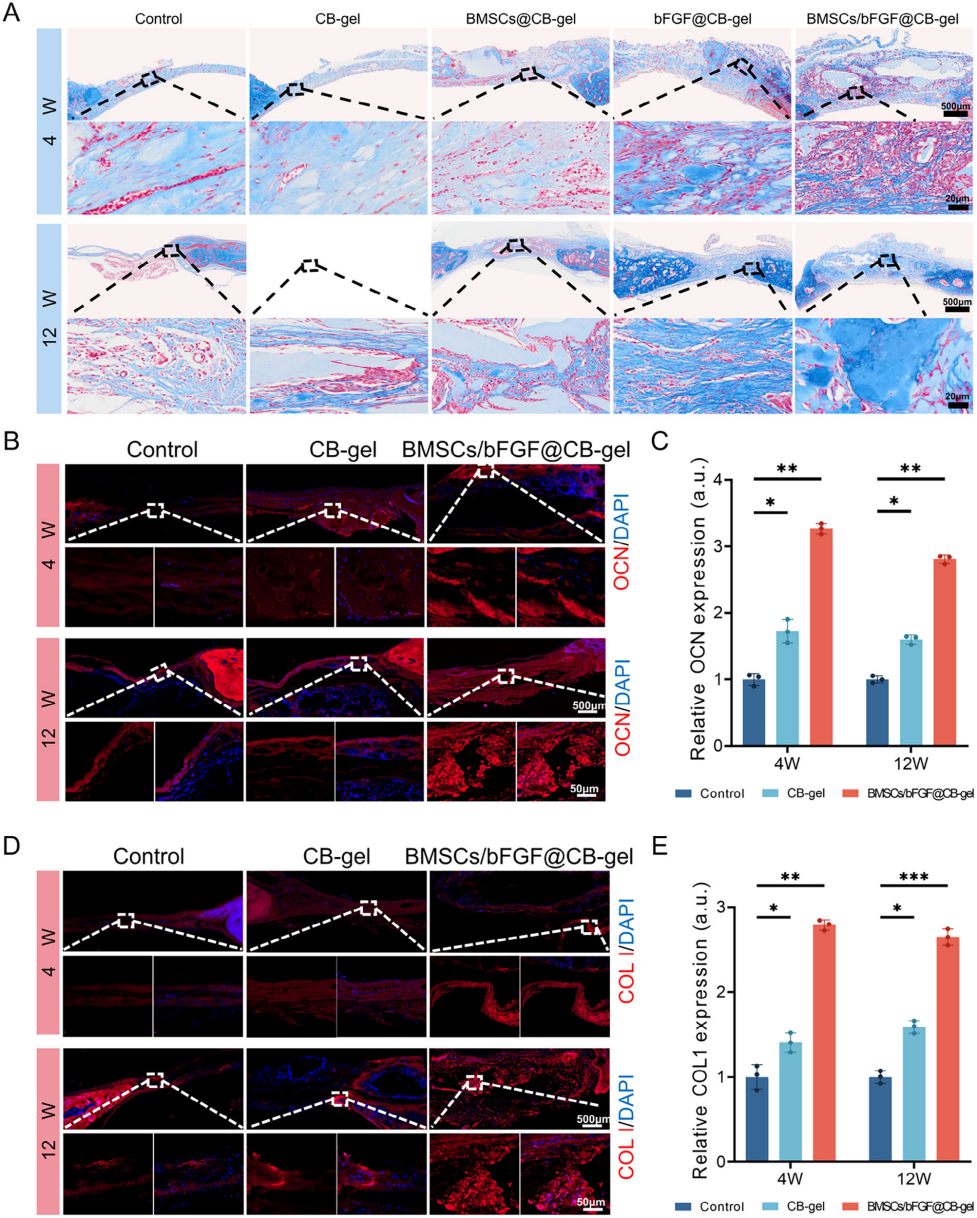
bFGF@CB‐gel–mediated BMSCs delivery increases the expression of osteogenesis‐related markers in vivo. (A). Masson's trichrome staining 4 and 12 weeks after surgery in cranial defects. (B,C). Immunofluorescence staining of OCN(B) and COL1(C) at 4 and 12 weeks after surgery in a cranial defect model. (D,E). Quantitative analysis of OCN(D) and COL1(E) immunofluorescence at 4 and 12 weeks after surgery in cranial defects. The data are presented as mean ± SD, *n* = 3. ^*^ *p <* 0.05, ^**^ *p <* 0.01 and ^***^ *p <* 0.001.

Analysis at 12 weeks post‐implantation revealed further progression of bone formation compared with 4 weeks. Micro‐CT reconstruction showed a larger amount of mineralized tissue and more continuous bony structures in the BMSCs/bFGF@CB‐gel group than in the other groups (Figure 6C), accompanied by increased BV/TV and trabecular number (Tb.N) compared to 4‐week results (Figure 6D). However, consistent with the characteristics of a critical‐sized cranial defect model, complete bridging of the defect was not uniformly observed histologically in all samples. H&E staining indicated that newly formed bone exhibited a more mature lamellar‐like appearance at 12 weeks compared with the early woven‐bone stage at 4 weeks, while residual connective tissue could still be found in the central region of the defect (Figure 6E). Masson's trichrome staining (Figure 8A) and immunofluorescence for osteocalcin (OCN) and type I collagen (COL I) (Figure 8B–E) further supported the time‐dependent maturation of the osteogenic matrix. Taken together, these findings indicate that the bFGF@CB‐gel scaffold substantially accelerates bone regeneration and promotes maturation of new bone, but full defect closure may require a longer healing time or additional osteoconductive/space‐maintaining reinforcement.

In summary, the bFGF‐loaded CB composite hydrogel efficiently distributes BMSCs and synergistically promotes angiogenesis and osteogenesis, enabling coordinated bone regeneration. Its pronounced long‐term efficacy identifies it as a solid biomaterial platform for successful bone lesion repair.

## 3 Discussion

CSD poses a major clinical challenge due to intertwined barriers of mechanical insufficiency, osteogenic deficit, and impaired angiogenesis. Current bone tissue engineering increasingly emphasizes rebuilding a pro‐regenerative niche that supports cell retention and survival, delivers instructive biochemical cues, and enables vascular‐bone crosstalk to sustain osteogenesis [54, 55]. Here, we developed a dual‐network bioactive scaffold, bFGF@CB‐gel, which synergistically integrates biomechanical support, molecular signaling, and cellular activity. The system leverages a photocurable ChSMA mineralized scaffold for mechanical stability and ECM‐mimicking BMSCs delivery, a bio‐nano BC carrier for sustained bFGF release (activating EGFL/Itga2b adhesion and COMP/PI3K/AKT osteogenic), and bFGF‐mediated microvascular endothelial cell migration and angiogenesis (PI3K/AKT/eNOS pathways). In a rat CSD model, bFGF@CB‐gel significantly enhanced new bone volume and vascular density, outperforming simplistic function‐stacking designs. This platform offers a promising local delivery system for reconstructive surgery, bridging mechanistic insights with clinical needs.

Conceptually, the advance of bFGF@CB‐gel is not simply the co‐presence of a growth factor and stem cells, but a material‐enabled strategy that builds on prior efforts to couple osteogenesis with angiogenesis in bone regeneration [54, 55, 56] and to enhance vascularized repair through bioactive/photocrosslinked platforms [57, 58, 59, 60]. In parallel, our material selection is grounded in established evidence that glycosaminoglycan‐based matrices support extracellular matrix regulation and bone regeneration‐relevant cellular behaviors [38, 61, 62, 63], while bacterial cellulose (BC) offers a nanofibrous ECM‐like architecture, high crystallinity, and robust mechanics that make it attractive for bone‐regenerative composites and cell‐instructive scaffolds [45, 46, 48, 64]. Against this background, our design addresses two translational pain points in parallel: (i) insufficient cell‐matrix interactions that limit BMSCs engraftment in hostile post‐implantation environments [65], and (ii) the instability/high diffusibility of bFGF that can cause burst loss and often motivates auxiliary affinity ligands, micro/nanocarriers, or covalent immobilization [36, 37]. By interpenetrating a covalently crosslinked ChSMA network with a BC nanofiber‐derived physical network, CB‐gel provides ECM‐mimetic topographical cues and reinforced mechanics while maintaining rapid photocuring and injectability. Importantly, BC simultaneously serves as an intrinsic, carrier‐free reservoir that stabilizes and sustains bFGF presentation, helping preserve a localized trophic microenvironment without introducing additional affinity modifiers or particulate carriers that may complicate formulation or compromise cytocompatibility [36, 37]. These structure‐function couplings help explain why CB‐gel improved BMSCs spreading and proliferation in vitro and potentiated the downstream osteogenic program after implantation.

From a materials‐healing timeline perspective, an ideal scaffold should provide early protection and instructive signaling, then gradually yield space for tissue ingrowth and remodeling. We therefore emphasize that CB‐gel primarily serves as an early‐stage delivery depot and pro‐regenerative niche that supports BMSCs activation and initiates angiogenesis and osteogenic commitment, after which material resorption facilitates host tissue invasion and matrix remodeling. In our model, the hydrogel appeared to be substantially degraded in vivo by week 4 based on gross observation and histology, although residual gel volume or mass was not quantitatively measured. Meanwhile, histology indicates that defect bridging was still ongoing at 4 and 12 weeks, with new bone forming progressively from the margins toward the center‐consistent with the staged nature of bone healing in critical‐sized defects. Thus, early microenvironmental support and later‐stage bone maturation are not contradictory; instead, they reflect sequential phases of regeneration. Future tuning of crosslinking density and network architecture could extend residence time when longer‐term space maintenance is desired.

Mechanistically, transcriptomic profiling identified PI3K/AKT signaling as a central hub linking ECM‐receptor interactions to osteogenic regulation. RNA‐seq revealed coordinated upregulation of ECM‐related genes (e.g., Col6a1/2 and Adamts5/9/15) together with pro‐angiogenic cues (e.g., Angpt1 and Pdgfra), and enrichment analyses highlighted extracellular matrix remodeling and myeloid proliferation as prominent processes. PI3K/AKT signaling emerged as a dominant axis connecting ECM cues with broader metabolic programs, and pathway activation was supported by Western blotting. Notably, RNA‐seq further suggested a hydrogel‐context adhesion‐proliferation module, which is particularly relevant to cell‐laden biomaterials where cell anchorage and mechanotransduction govern survival and fate [66]. Consistently, key adhesion‐related nodes (e.g., Itga8, Itga2b, and EGFL6) were validated at the protein level. Importantly, pharmacological PI3K inhibition suppressed AKT phosphorylation and attenuated the bFGF@CB‐gel‐induced upregulation of adhesion‐associated proteins and representative osteogenic outputs, providing inhibitory evidence that the pro‐osteogenic effects of bFGF@CB‐gel are, at least in part, PI3K/AKT dependent.

Angiogenesis is a rate‐limiting step for bone repair because perfusion restores oxygen/nutrient delivery and enables pro‐osteogenic vascular signaling [7, 8]. Building on prior strategies that couple angiogenic stimulation with osteogenesis [54, 58], we strengthened the evidence chain for the pro‐angiogenic capacity of bFGF@CB‐gel by integrating functional assays (migration and Matrigel tube formation) with expanded molecular readouts [17, 67, 68]. In vitro, bFGF@CB‐gel enhanced endothelial migration and promoted more extensive tube‐like network formation, accompanied by elevated expression of angiogenesis‐related markers. In vivo, immunostaining confirmed increased CD31 and VEGF at 4 weeks and further supported an enhanced pro‐angiogenic microenvironment via additional HIF‐1α and EMCN staining, consistent with improved formation of specialized pro‐osteogenic microvessels [7, 8]. Given that bFGF is a well‐recognized pro‐angiogenic factor with established roles in endothelial activation and neovascularization [67, 68, 69], this angiogenic support likely synergizes with BMSCs‐driven osteogenesis to accelerate mineralized tissue formation in the defect.

Despite these encouraging results, several limitations merit discussion. First, quantitative tracking of in vivo residual material (e.g., imaging‐based volume analysis or label‐enabled longitudinal monitoring) was not performed, and incorporating such measurements would enable a more rigorous alignment of degradation kinetics with the staged regenerative timeline. Second, although PI3K inhibition and protein‐level analyses support a PI3K/AKT‐dependent adhesion‐osteogenesis program, deeper causal mapping remains warranted, including perturbation of specific downstream nodes (e.g., integrin‐related components or AP‐1 modules) and cell‐type‐resolved interrogation to delineate BMSCs‐intrinsic versus endothelial/host contributions. Third, the interactions between transplanted BMSCs and host immune components (e.g., macrophage polarization) were not systematically profiled; future work integrating immunomodulatory cues may further optimize healing. Finally, longer follow‐up and large‐animal studies will be valuable to evaluate defect bridging, remodeling, and translational robustness in anatomically and mechanically more demanding settings.

## 4 Conclusion

In this study, we developed a dual‐network bioactive scaffold, bFGF@CB‐gel, which synergistically integrates biomechanical support, molecular signaling, and cellular activity to address key clinical challenges in CSD repair, including mechanical insufficiency, osteogenic deficit, and impaired angiogenesis. The system leverages a photocurable ChSMA mineralized scaffold for mechanical stability and ECM‐mimicking BMSCs delivery, a bio‐nano BC carrier for sustained bFGF release (activating EGFL/Itga2b adhesion and COMP/PI3K/AKT osteogenic), and bFGF‐mediated microvascular endothelial cell migration and angiogenesis (PI3K/AKT/eNOS pathways). In a rat CSD model, bFGF@CB‐gel significantly enhanced new bone volume and vascular density, outperforming simplistic function‐stacking designs. This work establishes a rational, synergistic paradigm for CSD repair, moving beyond single‐target strategies to orchestrate multi‐pathway regeneration. Its translational potential offers a promising local delivery system for reconstructive surgery, bridging mechanistic insights with clinical needs.

## Author Contributions

Y.F., B. C., Y. Xu., and X. Xu. designed and synthesized the multifunctional bioactive scaffold under the guidance of W.Z. and S.H., and conducted in vitro and in vivo experiments. B.B., B.H., B.C., C.L., and T.L. provided assistance. Under W.Z. and S.H. guidance, Y.F. analyzed the data and wrote the manuscript. W.Z. and S.H. provided guidance at all stages of the project. All authors contributed to the completion of the manuscript. All a uthors have approved the final version of the manuscript.

## Conflicts of Interest

The authors declare no conflicts of interest.

## Supporting information




**Supporting File**: advs74913‐sup‐0001‐SuppMat.docx.

## Data Availability

The data are not publicly available due to privacy or ethical restrictions.
