# Supplementary material for: Multifunctional Bioactive Scaffold Facilitating BMSCs‐Driven Osteogenesis and Vascularization in Critical‐Sized Bone Defect Repair
Source: Adv Sci (Weinh). 2026 Apr 9;13(31):e22692. doi: 10.1002/advs.202522692 (PMC13588067; doi:10.1002/advs.202522692)
Supplement: Supplementary file 1 [file ADVS-13-e22692-s001.docx]

Supporting Information

**Multifunctional Bioactive Scaffold Facilitating BMSCs-Driven Osteogenesis and Vascularization in Critical Sized Bone Defect Repair**

*Yunze Feng, Bingzhi Chen, Xin Xu, Yu Xu, Bingtao Hu, Tongbo Li, Dingxin Zhang, Baoshuai Bai,* *Wanglong Xu, Le Li, Wencan Zhang^*^, Haipeng Si^*^*

**Supporting figures**

Figure S1. **Characterisation of hydrogels.** A). Cross-section image of hydrogel (SEM). B). CCK-8 assay evaluating cell proliferation on hydrogels. C,D). Kinetic model fitting of bFGF release profiles. E). F-actin (phalloidin) staining, showing enhanced cell spreading on hydrogels. F,G). Western blot analysis and quantification of adhesion-related proteins. The data presented as mean ± SD, n = 3. **p < 0.05, **p < 0.01 and ***p < 0.001.*

Figure S2. **Cytocompatibility of hydrogels.** A). Live/dead assay of BMSCs co-cultured with hydrogels for 4 and 7 d. B). Quantification of cell viability by live/dead assay. The data presented as mean ± SD, n = 3. **p* < 0.05, ***p* < 0.01 and ****p* < 0.001.

Figure S3. **Optimal Concentrations of bFGF.** A,B). Immunofluorescence staining(A) and quantification(B) of RUNX2 in BMSCs after 7-day bFGF treatment. C). Quantification of cell viability by live/dead assay. D). CCK-8 assay analysis of BMSCs treated with bFGF for 4(C) and 7(D)d. The data presented as mean ± SD, n = 3. **p* < 0.05, ***p* < 0.01 and ****p* < 0.001.

Figure S4. **Cytocompatibility of hydrogels.** A).  H&E staining images of heart, liver, spleen, lung, and kidney tissues in each treatment group at 12 weeks. B). Serum biochemical parameters including liver function markers (ALT, AST, ALP) and renal function markers (UA, BUN, CR) in each treatment group at 12 weeks. n=3.

Figure S5. Multi-omics validation of the bone repair mechanism facilitated by the bFGF@CB-gel hydrogel system. A,B). GO terms for ECM remodeling. C,D). Western blot analysis and Quantitative analysis of PI3K 、AKT、p-PI3K、p-AKT. The data presented as mean ± SD, n = 3. **p < 0.05, **p < 0.01 and ***p < 0.001*.

Figure S6. bFGF@CB-gel hydrogel system stimulates the production of angiogenesis-related proteins in vivo. A,C). Immunofluorescence staining(A) and quantification(C) of HIF-1α at 4 w after surgery in calvarial defect. B,D). Immunofluorescence staining(B) and quantification(D) of EMCN at 4 w after surgery in calvarial defect. The data presented as mean ± SD, n = 3. **p < 0.05, **p < 0.01 and ***p < 0.001.*

Figure S7. Quantitative statistical analysis of residual hydrogel contentQuantitative statistical analysis of residual hydrogel content. The data presented as mean ± SD, n = 3*. *p < 0.05, **p < 0.01 and ***p < 0.001.*

**Table S1. The primer sequences used in these experiments.**

| Table S1. The primer sequences used in these experiments. | | |
| --- | --- | --- |
| Geen name | Forward (5' to 3') | Reverse (5' to 3') |
| *β-actin* | GGCTCTTGTGCGTACTGTCCTTC | CTTGGTGTCGGCTGGTCAGATG |
| *ALP* | AGTGTGGCAGTGGTATTGTAGG | CACACACAAAGCACTCGGGG |
| *RUNX2* | ACGAATGCACTATCCAGCCA | GCAGGTACGTGTGGTAGTGA |
| *OPN* | GGTGATAGCTTGGCTTACGGA | TGGCATCGGGATACTGTTCA |

**Experimental Section**

*Materials and Characterization*

*Materials and reagents*

ChSMA (EFL-ChSMA-001, Mw: 65 ∼85 kDa, DS: 25%) and Lap photoinitiator were obtained from Suzhou Intelligent Manufac- turing Research Institute Co., Ltd.(Suzhou, China). The Cell Counting Kit-8 (CCK-8) were obtained from Shanghai Beyotime Co.,Ltd.(Shanghai, China). The trypsin, penicillin-streptomycin solution, and Dulbecco’s Modified Eagle Medium/Nutrient Mixture F-12 (DMEM/F12) were obtained from Wuhan Saiweier Biotechnology Co., Ltd.(Wuhan, China). The fetal bovine serum (FBS) were obtained from Shanghai CellMax Biotechnology Co., Ltd.(Shanghai, China), while the Alizarin Red S (ARS) Staining Kit, BCIP/NBT Alkaline Phosphatase Color Development Kit, and BCA Protein Quantification Kit were all obtained from Shanghai Biyuntian Biotechnology Co., Ltd(Shanghai, China).

*Hydrogel Synthesis*

To prepare the composite hydrogel CB, 25 mg of LAP was dissolved in 10 mL of PBS buffer in a brown bottle and heated in a water bath at 40–50°C for 15 minutes with intermittent shaking until fully dissolved, yielding a 0.25% (w/v) LAP initiator standard solution. Subsequently, 0.5 g of ChSMA was mixed with 10 mL of the LAP initiator solution and dissolved in the dark at room temperature for 30 minutes under periodic agitation. The mixture was then sterilized through a 0.22 μm sterile syringe filter to obtain a 10% (w/v) ChSMA-001 solution. Finally, ChSMA-001 and BC were combined at varying volume ratios and exposed to a 405 nm ultraviolet light source for 10–30 seconds to induce gelation, resulting in the formation of the composite hydrogel CB.

*Scanning electron microscopy (SEM)*

Scanning electron microscope (SEM, Zeiss, China) was used to observe the surface and internal structure of the hydrogel. The steps were as follows: the surface and internal structure of the lyophilized hydrogel samples were sputter-coated with gold-palladium alloy for 3 min to improve the electrical conductivity of the samples. Subsequently, SEM images were acquired at different magnifications to analyze the microscopic morphology.

***Fourier Transform Infrared Spectroscopy (FTIR)***

Solid samples (CB-gel) were prepared by pressing potassium bromide directly in the sample holder; the instrument was warmed up and calibrated for background signal. During the test, the wave number range (4000-400 cm^-^¹), resolution (4 cm^-^¹), and number of scans (32) were set, and the absorption spectral data were collected. Precautions include sample drying to avoid interference from water peaks, and complex mixtures need to be further validated in combination with other techniques.

*Compressive mechanical properties*

The compressive mechanical properties of different hydrogels were tested using an electronic universal testing machine and a 5 Nm (N) transducer. The Young's modulus of each hydrogel was calculated from the slope of the stress-strain curve in the range of 0-10% of the strain interval.

*Swelling test*

Hydrogel samples dried to constant weight were immersed in PBS, periodically removed, swabbed dry and weighed (Wt), and the initial dry weight (W0) was recorded. Dissolution rates$\frac{Wt-W0}{W0}\times100\%$ were calculated from time-point data, and each set of experiments was repeated three times. Key control parameters include a constant temperature environment (e.g., 37°C), strict time points, and precision weighing. Care should be taken to ensure that samples are uniform in size, surfaces are gently wiped, and liquids are changed to avoid contamination during prolonged testing.

*Cell culture*

BMSCs were extracted from the femur and tibia of Sprague-Dawley rats using the methodology outlined in the literature^[4]^. The procedure was as follows: five healthy 1-week-old male SD rats were selected, euthanized, and sterilized by soaking in 75% alcohol for 5 minutes; the muscle and skin tissues of both hind limbs were excised under aseptic conditions to expose the femur and tibia; the bone marrow cavities were repeatedly rinsed with a syringe to aspirate the DMEM/F12 medium in order to collect the bone marrow cells completely; the obtained cells were placed in an incubator at 37°C with 5% CO₂. The cells were grown in DMEM/F12 complete medium (DMEM/F12 supplemented with 10% fetal bovine serum and 1% penicillin-streptomycin) in a 37°C, 5% CO₂ incubator, with medium changes every 3 days. The cells were passaged when the degree of confluence reached 90%, and the third-generation BMSCs were selected for subsequent osteogenesis-related experiments (osteogenic induction medium: DMEM/F12 supplemented with 10% fetal bovine serum, 1% penicillin-streptomycin, 0.2% ascorbic acid, 1% β-glycerophosphate, and 0.01% dexamethasone). The entire process should be properly maintained in a sterile atmosphere to assure the quality of cell culture. Rat Brain microvascular endothelial cells (BMECs) were likewise placed in a 37°C, 5% CO₂ incubator and grown in DMEM/F12 complete media (DMEM/F12 with 10% fetal bovine serum and 1% penicillin-streptomycin), with medium changes every 3 days. The cells were passaged until the degree of confluence reached 90%, and the third-generation BMECs were selected for additional research.

*Preparation of BMSCs-laden hydrogels*

The composite hydrogel bFGF@CB-gel encapsulating BMSCs was fabricated following the procedure described in Section 2.2 with modifications: Prior to ultraviolet (UV)-induced photopolymerization, BMSCs (passage 3–5) and basic fibroblast growth factor (bFGF, 10 ug/mL) were homogenously encapsulated into the CB prepolymer soluvalue was measured. As for the detection of cell activity, the instructions for the live/dead cell staining kit were followed, and the images were finally obtained by fluorescence microscopy.

*Alkaline phosphatase staining.*

The hydrogels loaded with cells were cultured in an osteogenic induction medium for up to 7 and 14 days, and for observation of ALP expression, the cell-hydrogel complexes were washed with PBS and analyzed by ALP staining directly, and the ALP-stained images were captured by light microscopy.

*Alizarin red staining*

The cell-loaded hydrogels were cultured in an osteogenic induction medium until 7 and 14 days and then sequentially subjected to the following treatments: first, the cell-hydrogel complexes were washed three times with PBS and fixed in 4% paraformaldehyde for 30 min; subsequently, the cell-hydrogel complexes were stained using alizarin red S (ARS) staining solution, and the images of the mineralized nodules were captured by light microscopy.

*Western blot analysis*

BMSCs and BMECs were lysed in RIPA lysis buffer (Beyotime, China) containing protease inhibitor (Beyotime, China) and phosphatase inhibitor (Beyotime, China). A BCA protein assay kit (Solarbio, China) was used to measure the protein concentration. Each protein suspension after denaturation by SDS loading buffer was separated on a 10% or 6% SDS-PAGE gel (Beyotime, China) and then transferred onto 0.22μm or 0.45 μm polyvinylidene fluoride membranes (PVDF, Invitrogen, the US). Then, the PVDF membranes were blocked with 5% BSA (bovine serum albumin, Sigma, Germany) for 2 hours and incubated with primary antibodies at four °C overnight. Secondary antibodies were added for 1 hour. An ultra-sensitive enhanced chemiluminescent (Invitrogen, the US) was used for protein detection by the ChemiDoc MP Imaging System (Bio-Rad, the US). The protein blot analysis was performed using Image J software. The primary antibodies used included: rabbit-anti-RUNX2(20700-1-AP, Proteintech, the US), mouse-anti-BMP-2(66383-1-lg, Proteintech, the US), rabbit-anti-OPN(22952-1-AP, Proteintech, the US), rabbit-anti-CD31(11265-1-AP, Proteintech, the US), rabbit-anti-VEGF(26415-1-AP, Proteintech, the US), rabbit-anti-eNOS(27120-1-AP, Proteintech, the US), rabbit-anti-COMP(28369-1-AP, Proteintech, the US), rabbit-anti-EGFL6(24748-1-AP, Proteintech, the US), rabbit-anti-Itga2b(24552-1-AP, Proteintech, the US), rabbit-anti-Itga8(29775-1-AP, Proteintech, the US), rabbit-anti-PI3K(20584-1-AP, Proteintech, the US), rabbit-anti-AKT(10176-2-AP, Proteintech, the US), rabbit-anti-GADPH(10494-1-AP, Proteintech, the US) and mouse-anti-Beta Actin(66009-1-Ig, Proteintech, the US). The secondary antibodies used included: HRP-conjugated Affinipure Goat Anti-Mouse IgG(H+L) (SA00001-1, Proteintech, the US), HRP-conjugated Affinipure Goat Anti-Rabbit IgG(H+L) (SA00001-2, Proteintech, the US).

*qRT-PCR assays*

In the experiments, all groups were prepared according to the method described in section 2.8, where BMSCs were first cultured for a period of 7 and 14 days, respectively, and then were suspended in TRIzol (Invitrogen, the US), and a total RNA extraction kit (Solarbio, China) was used to harvest RNA. The RNA amount and purity were quantified by NanoDrop ND-1000 (NanoDrop, the US). Subsequently, the PrimeScript^TM^ RT reagent kit (Takara, Japan) was used to reverse the mRNA into cDNAs. Afterward, Real-Time Quantitative Polymerase Chain Reaction (RT-qPCR) was performed using LightCycler 960 with TB Green® Premix Ex Taq™ II (Tli RNaseH Plus) (Takara, Japan). The primer sequences used in these experiments are shown in Table S1, Supporting Information.

***BMECs Scratch Wound Healing Assay***

BMECs were maintained in endothelial cell growth medium (EGM-2) containing 5% fetal bovine serum and seeded into 6-well plates until reaching 90–100% confluence. A linear wound was then generated in the cell monolayer using a sterile 200 μL pipette tip. After washing twice with PBS to remove detached cells, fresh medium was added and the cells were incubated at 37°C with 5% CO₂. Images of the scratched area were captured at 0, 12, 24 and 48 h using an inverted microscope. The wound healing area was quantified with ImageJ software, and the percentage of wound closure was calculated to evaluate cell migratory capacity.

***BMECs Tube Formation Assay on Matrigel***

BMECs were cultured in endothelial cell growth medium (EGM-2) supplemented with 5% fetal bovine serum. Growth factor-reduced Matrigel (Corning) was thawed on ice, and 50 μL was pipetted into each well of a pre-chilled 96-well plate, followed by incubation at 37°C for 30 min to polymerize. BMECs (passage 3–5, 1×10⁴ cells/well) were seeded onto the Matrigel-coated surface in EGM-2. After 12 h of incubation at 37°C/5% CO₂, tube formation was visualized under an inverted microscope, and images were captured for morphometric analysis (ImageJ software) to quantify total tube length and node number.

*Immunofluorescence Staining*

***Cell Immunofluorescence Staining Protocol***

The 4% PFA (paraformaldehyde, Solarbio, China) was used to fix the cells for 30 min. Then, the cells were blocked and permeabilizated ) for 1 hour by 5% BSA with 0.3% Triton X-100 (Sigma, Germany. The samples were incubated with primary antibodies at four °C overnight and secondary antibodies for two hopur. Finally, the cells were stained with DAPI (Solarbio, China) for 10 min. CLSM was used to observe all samples. The primary antibodies included: rabbit-anti-RUNX2(20700-1-AP, Proteintech, the US), mouse-anti-BMP-2(66383-1-lg, Proteintech, the US), rabbit-anti-CD31(11265-1-AP, Proteintech, the US), rabbit-anti-VEGF(26415-1-AP, Proteintech, the US), rabbit-anti-EMCN(11828-1-AP, Proteintech, the US) and rabbit-anti-HIF1A(20960-1-AP, Proteintech, the US). The secondary antibodies included: CoraLite488-conjugated Goat Anti-Mouse IgG(H+L) (SA00013-1, Proteintech, the US), CoraLite594-conjugated Goat Anti-Rabbit IgG(H+L) (SA00013-4, Proteintech, the US), Donkey Anti-Goat IgG H&L (Cy3 ®) (ab6949, Abcam, England).

***Tissue sections Immunofluorescence Staining Protocol***

At 4 and 12 weeks post-implantation, calvarial defect tissues were harvested, fixed, decalcified, embedded in paraffin, and sectioned for immunofluorescence staining. Osteogenic markers such as COI-1 and Osteocalcin were detected at both time points, while angiogenic markers such as CD31 were assessed at 12 weeks. Cell nuclei were counterstained with DAPI, and fluorescence signals were observed and analyzed under a fluorescence microscope. The primary antibodies included: mouse-anti-COI-1(67288-1-Ig, Proteintech, the US), rabbit-anti-OCN(23418-1-AP,Proteintech, the US), rabbit-anti-CD31(11265-1-AP, Proteintech, the US), rabbit-anti-VEGF(26415-1-AP, Proteintech, the US), The secondary antibodies included: CoraLite488-conjugated Goat Anti-Mouse IgG(H+L) (SA00013-1, Proteintech, the US), CoraLite594-conjugated Goat Anti-Rabbit IgG(H+L) (SA00013-4, Proteintech, the US), Donkey Anti-Goat IgG H&L (Cy3 ®) (ab6949, Abcam, England).

***In vivo calvarial defect model***

All animal experiments in this study were conducted in strict accordance with the Institutional Guidelines for the Care and Use of Laboratory Animals of Shandong University and were approved by the Animal Ethics Committee of Shandong University (Approval No. 23078). In this study, a hydrogel loaded with allogeneic BMSCs was used to evaluate its osteogenic efficacy in vivo using a critical bone defect model in rats (n=40 rats, 12 weeks old). After anesthesia with isoflurane, a sagittal incision was made along the midline of the skull. A circular drill was used to create bilateral 5 mm bone defects in the skull, which were then irrigated with physiologic saline. Subsequently, four groups of implant materials were implanted: CB-gel, B@CB-gel, bFGF@CB-gel and BMSCs/bFGF@CB-gel (n=8 per group), with BMSCs at a density of 1×10⁶ cells/ml, then the wound was closed with layered sutures. The rats were observed continuously until 4/12 weeks after surgery. Then they were euthanized under excessive inhalation of isoflurane and four samples were taken from each group for histologic and micro-CT analysis.

***Imaging analysis***

In the experiment, the rats in each group were euthanized at 4 and 12 weeks postoperatively, and cranial samples were collected. The samples were examined using micro-computed tomography (micro-CT), including the initial scanning of the samples and the processing of the data using three-dimensional surface reconstruction techniques. On the basis of defining the defect area, key parameters such as bone volume fraction (BV/TV) were quantitatively analyzed using micro-CT analysis software.

***Histological staining***

In the experiment, the rats in each group were euthanized at 4 and 12 weeks postoperatively, and cranial samples were collected, which were fixed, decalcified, embedded, and then stained with hematoxylin-eosin (HE) and Masson's trichrome(Masson), respectively.

***Statistical analysis***

All statistical analyses were carried out using GraphPad Prism (version 9.5; GraphPad Software, USA). Unless otherwise stated, all experiments were performed with n = 3 independent samples per group, and data are presented as mean ± standard deviation (mean ± SD). Raw data were inspected for obvious recording or processing errors, and no data points were excluded from statistical analyses unless explicitly stated.

For comparisons among multiple groups, one-way ANOVA was applied for single-factor designs and two-way ANOVA for experiments involving two independent variables, followed by Tukey’s multiple-comparisons post hoc test. All tests were two-sided, and statistical significance was defined as P < 0.05 (α = 0.05). The specific statistical test and sample size (n) for each analysis are provided in the corresponding figure legends.
